# Effect of medication on the rostrolateral prefrontal oxygenation and thalamic volume asymmetry in youths with ADHD

**DOI:** 10.3389/fnint.2025.1591465

**Published:** 2025-05-21

**Authors:** Hyuna Kim, Dahyun Kang, Yong Hun Jang, Ja-Hye Ahn, Sojin Won, Hyun Ju Lee, Johanna Inhyang Kim

**Affiliations:** ^1^Department of Translational Medicine, Hanyang University Graduate School of Biomedical Science and Engineering, Seoul, Republic of Korea; ^2^Department of Psychological Sciences, University of Missouri, Columbia, United States; ^3^Department of Pediatrics, Hanyang University Hospital, Hanyang University College of Medicine, Seoul, Republic of Korea; ^4^Hanyang Inclusive Clinic for Developmental Disorders, Seoul, Republic of Korea; ^5^Institute of Mental Health, Hanyang University, Seoul, Republic of Korea; ^6^Department of Psychiatry, Hanyang University Hospital, Hanyang University College of Medicine, Seoul, Republic of Korea

**Keywords:** ADHD, fNIRS, sMRI, Stroop test, rostrolateral prefrontal, asymmetry

## Abstract

**Introduction:**

Symptoms of attention-deficit/hyperactivity disorder (ADHD) are closely associated with impaired executive function. Medication is the first-line treatment for ADHD, yet its effects on brain function and structure remain unclear. To investigate medication-related brain alterations in children with ADHD, we used functional near-infrared spectroscopy, which captures cortical hemodynamic activity, and structural magnetic resonance imaging, which measures subcortical volume.

**Methods:**

We investigated the differences in brain hemodynamic activity between 23 children with ADHD taking medication and 22 children who were not taking medication.

**Results:**

Compared with the medicated ADHD group, the unmedicated ADHD group showed significantly reduced activation in the left rostrolateral prefrontal cortex (channel 9, *p* = 0.01; channel 13, *p* = 0.02) and dorsolateral prefrontal cortex (channel 14, *p* = 0.01). The unmedicated group also exhibited a negative correlation between oxygenated hemoglobin and symptom severity, whereas the medicated group showed a positive correlation. Furthermore, abnormal asymmetry of the thalamic volume was reduced in the medicated group compared to the unmedicated group.

**Discussion:**

These findings suggest that increased prefrontal activation and reduced thalamic asymmetry may reflect medication-related improvements in inhibitory control in children with ADHD.

## 1 Introduction

Attention-deficit hyperactivity disorder (ADHD) is one of the most common neurodevelopmental disorders in school-age children and is characterized by age-inappropriate symptoms of inattention, hyperactivity, and impulsivity (American Psychiatric Association, 2013). According to a recent systematic review and meta-analysis, the prevalence of ADHD in children under the age of 12 is approximately 7.6% (Salari et al., 2023). Approximately 43% of individuals with these symptoms progress to a chronic state during adulthood (Di Lorenzo et al., 2021), debilitating the quality of their social, academic, or occupational functions (Gjervan et al., 2012; Holst and Thorell, 2020). Furthermore, patients with ADHD show impaired response inhibition, which is associated with the suppression of executive function, causing crucial neurophysiological defect (Johnstone et al., 2013; van Rooij et al., 2015; Breitling-Ziegler et al., 2020).

The first-line treatment for ADHD is medication, including methylphenidate (MPH; a dopamine and norepinephrine reuptake inhibitor) and atomoxetine (ATM; a selective norepinephrine reuptake inhibitor). However, the neurobiological effects of medication in ADHD are not fully understood, particularly regarding how medication modifies brain function and structure. Therefore, there is a growing need for objective neuroimaging methods that can assess treatment effects beyond conventional clinician-administered or patient-reported assessments. Functional near-infrared spectroscopy (fNIRS) is a non-invasive, portable neuroimaging technique with superior spatial resolution and reduced susceptibility to motion artifacts compared to other modalities (Cui et al., 2011; Sutoko et al., 2020). fNIRS has been increasingly used to measure regional brain oxygenation and has shown promise for diagnosing ADHD and monitoring treatment effects. A previous study suggested that reduced prefrontal activation measured by fNIRS could serve as an objective biomarker for predicting clinical course and treatment response (Yukifumi et al., 2017).

Research using fNIRS has demonstrated discriminative signals in specific brain regions, both between children with ADHD and controls and between medication-naïve and non-naïve children with ADHD (Kaga et al., 2020). However, a previous meta-analysis reported inconsistencies among study results: some studies showed hyperactivation in the dorsolateral prefrontal cortex and left superior frontal cortex (Jourdan Moser et al., 2009; Tsujimoto et al., 2013; Nagashima et al., 2014b; Suzuki et al., 2017), whereas others reported hypoactivation in the right prefrontal regions (Gosse et al., 2022). Notably, reduced oxyhemoglobin signals in the inferior and middle frontal gyri during cognitive tasks were normalized following medication treatment (Nagashima et al., 2014a), suggesting that fNIRS may be sensitive to functional brain changes associated with medication in ADHD. Further studies have shown that children with ADHD receiving medication exhibited greater prefrontal cortex (PFC) activation during high-workload tasks compared to unmedicated children with ADHD, although no significant differences were observed in cognitive task performance (Matsuura et al., 2014). Nagashima et al. additionally reported that the normalization pattern of brain activation in medicated children was asymmetrical across the frontoparietal attention network (Nagashima et al., 2014b). Moreover, administration of atomoxetine (Nagashima et al., 2014a) and methylphenidate (Monden et al., 2012a) was associated with increased activation in the right middle frontal gyrus and right inferior prefrontal gyrus. In contrast, other studies have reported an opposite pattern, showing hyperactivation particularly in the left PFC during the Stroop Color-Word Test (SCWT), regardless of medication type (Nakanishi et al., 2017).

Although fNIRS provides valuable information regarding cortical hemodynamic changes, it cannot assess subcortical structures that are crucial in ADHD pathophysiology. Conversely, sMRI can capture structural alterations in deep brain regions, such as the thalamus, which are implicated in ADHD symptom expression and treatment response. Therefore, integrating fNIRS and sMRI enables a more comprehensive understanding of both functional and structural brain alterations associated with medication effects in children with ADHD. Particularly, hemispheric differences in brain structure are thought to reflect aberrant developmental synchrony between the left and right brain in an asymmetric manner (Doi and Shinohara, 2017), contributing to or accompanied by ADHD symptom severity. A recent meta-analysis of abnormal hemispheric asymmetry using resting-state fMRI reported that children with ADHD had both structural (in the prefrontal cortex, subcortical cortices, and cerebellum) and functional abnormalities (in the inferior frontal gyrus, temporal pole, and cerebellum) (He et al., 2022). Particularly, as the symptoms of ADHD become severe, hemispheric brain asymmetry differences increase, indicating a pathoplastic role for asymmetry with a structural basis during development. Moreover, volumetric anomalies of thalamocortical regions have been demonstrated in children with ADHD, revealing the pathogenesis of various ADHD symptoms (Batty et al., 2015). Ivanov et al. showed reduced pulvinar volumes of the thalamus in patients with ADHD without medication compared to those with medication, suggesting a medication effect on the thalamic subcircuit (Ivanov et al., 2010). Subcortical changes in children with ADHD could be more evident in thalamic regions that could not be detected using fNIRS. Although fNIRS can provide information only on cortical activity, information on the thalamic volume in structural magnetic resonance imaging (sMRI) may help reveal the pathological characteristics of patients with ADHD.

Past studies have focused on comparing the hemodynamic response between children with ADHD receiving medication and controls, while there has been limited research comparing children with ADHD receiving medication and those not receiving medication. Moreover, numerous studies examining the neural substrates of the effects of medication have largely focused on single-modality neuroimaging or neurophysiological results. To address these limitations, the present study aimed to simultaneously examine the brain hemodynamic activity and structural asymmetry using fNIRS and sMRI in children with ADHD, both with and without medication. We hypothesized that children with ADHD receiving medication would show enhanced prefrontal activation and reduced abnormal thalamic asymmetry compared to unmedicated children with ADHD. By integrating multimodal neuroimaging techniques, this study provides novel insights into the neural mechanisms underlying the effects of ADHD medication in children, offering clinically meaningful biomarkers for monitoring treatment response and improving the reliability of functional brain activity measurements in this population.

## 2 Methods

### 2.1 Study design and participants

This study was a cross-sectional observational study designed to examine neurobiological differences between medicated and unmedicated individuals with ADHD at a single time point. As an observational, non-interventional study, clinical trial registration was not required under institutional or national guidelines. Participants were recruited from the psychiatric outpatient clinic at Hanyang University Medical Center and through online advertisements. Recruitment and data collection were conducted between May 12, 2020, and January 12, 2022. A total of 47 participants diagnosed with ADHD were initially enrolled. Two participants were excluded due to channel rejection during the fNIRS measurement, resulting in a final sample of 45 participants. Among these, 23 were assigned to the medicated group and 22 to the unmedicated group. All participants and their parents provided written informed consent to participate in the study after receiving sufficient explanation of the study. All experimental protocols were approved by the Institutional Review Board of the Hanyang University Medical Center (IRB Approval no. 2020-02-025) and performed in compliance with the principles of the Declaration of Helsinki.

The inclusion and exclusion criteria were identical for the medicated and unmedicated groups, except for medication status. Potential participants were included if they (1) were between 8 and 15 years of age; (2) fulfilled the ADHD diagnostic criteria of the Diagnostic and Statistical Manual of Mental Disorders fourth edition (DSM-5), confirmed by the Kiddie Schedule for Affective Disorders and Schizophrenia – Present and Lifetime version (K-SADS-PL) (Kaufman et al., 1997), and (3) their intelligence quotients (IQ) were above 70. Exclusion criteria included: (1) congenital hereditary problems; (2) brain injury such as cerebral palsy; (3) spasmodic disorder, other neurological disorders, or untreated dysesthesia (sensory disturbance); (4) past or current schizophrenia and childhood psychosis, autism spectrum disorder, or Tourette’s syndrome; (5) IQ scores under 70; and (6) comorbidity with obsessive-compulsive, depressive, or bipolar disorder.

The unmedicated ADHD group consisted of participants who had never received MPH or ATM treatment or had not taken medication within 4 weeks prior to participating in the study. The medicated group consisted of patients with ADHD whose dosages of methylphenidate or atomoxetine had not changed over the past month. The Clinical Global Impression Scale-Severity (CGI-S) score for both the drug-naïve and drug-medicated groups was > 4, indicating that their underlying symptoms were moderate to severe, and there was no remission. Among the medicated ADHD group, 17 participants took methylphenidate (mean dosage; 32.06 ± 19.78 mg), and 12 participants took atomoxetine (mean dosage 39.67 ± 14.67 mg). Among these participants, seven were prescribed methylphenidate and atomoxetine. The CGI scores for each group and drug information for the medication group are listed in Table 1. The diagnosis of psychiatric disorders was confirmed using the K-SADS-PL, which was conducted by a single board-certified psychiatrist. IQ scores were determined using the Korean version of the Wechsler Intelligence Scale for Children (WISC), Fourth or Fifth Edition (Kwan et al., 2011).

**Table 1:** Demographic and clinical characteristics.

Factors	Medicated ADHD (*n* = 23)	Unmedicated ADHD (*n* = 22)	*p*
Children characteristics			
Age (years)	11.09 ± 1.93	10.64 ± 1.73	0.42
Male sex	20 (87%)	14 (63.6%)	0.07
FSIQ	95.30 ± 16.70	94.14 ± 12.33	0.79
ADHD-RS-IV total	23.22 ± 7.50	28.27 ± 9.01	0.05
ADHD-RS-inattention	14.39 ± 4.55	16.36 ± 4.56	0.15
ADHD-RS-hyperactivity-impulsivity	8.83 ± 4.15	11.91 ± 5.08	0.04*
MPH	17		
MPH dose (mg)	32.06 ± 19.78		
ATM	12		
ATM dose (mg)	39.67 ± 14.67		
Height (cm)	153.69 ± 14.95		
Weight (kg)	51.52 ± 17.71		
Handedness			0.55
Left	2 (8.7%)	3 (13.6%)	
Right	20 (87.0%)	19 (86.4%)	
Mixed	1 (4.3%)	0 (0%)	
CGI			
CGI-S	4.22 ± 0.42	4.62 ± 0.59	0.02*
C-GAS	60.22 ± 7.46	56.00 ± 7.81	0.07
ATA visual task			
Omission error	57.83 ± 19.08	63.73 ± 19.70	0.31
Commission error	60.83 ± 18.09	63.55 ± 19.77	0.63
RT	62.70 ± 14.47	68.36 ± 12.99	0.18
RTV	57.30 ± 19.37	67.77 ± 17.39	0.03*
ATA auditory task			
Omission error	71.22 ± 23.27	67.77 ± 22.54	0.62
Commission error	69.74 ± 22.27	70.95 ± 22.90	0.86
RT	53.52 ± 8.87	53.50 ± 12.71	1.00
RTV	50.13 ± 10.76	53.50 ± 10.42	0.29
SCWT			
Word score	38.26 ± 10.18	39.32 ± 13.96	0.77
Color score	42.09 ± 8.86	44.14 ± 8.71	0.44
Color-word score	45.91 ± 10.89	46.45 ± 9.92	0.86
Interference score	57.26 ± 8.91	54.50 ± 10.76	0.35
Socioeconomic characteristics			
Economic Status			0.13
High	0 (0%)	1 (4.5%)	
High average	7 (30.4%)	3 (13.6%)	
Average	7 (30.4%)	14 (63.6%)	
Low Average	6 (26.1%)	2 (9.1%)	
Low	3 (13.0%)	2 (9.1%)	
Maternal education level			0.92
High school	2 (8.7%)	2 (9.5%)	
College or university	21 (91.3%)	19 (90.5%)	
Father’s education level			0.09
High school	5 (21.7%)	1 (4.5%)	
College or university	18 (78.3%)	21 (95.5%)	

Data are the mean ± SD or *n* (%). * Statistically significant at *p* < 0.05. ADHD, attention deficit/hyperactivity disorder; FSIQ, full-scale intellectual quotation; MPH, methylphenidate; ATM, atomoxetine; CGI-S, clinical global impression scale-severity; C-GAS, children’s global assessment scale; ATA, advanced test of attention; RT, response time; RTV, response time variability; SCWT, Stroop Color-Word Test. Six children were medicated with both MPH and ATM. * Statistically significant at *p* < 0.05. *Bold values indicate statistically significant differences at *p* < 0.05.

### 2.2 Clinical assessment tools

#### 2.2.1 ADHD Rating Scale

The ARS is the most widely used scale to screen children with ADHD by their parents and teachers and was developed by Du Paul based on the diagnostic criteria of ADHD illustrated in the DSM-5 (DuPaul et al., 2016). The scale consists of 18 items rated from 0 to 3, with parents or teachers scoring each item based on the frequency of the children’s problematic behaviors. The total scores of the odd-numbered questions measures inattention and the total number of even-numbered items measures impulsivity and hyperactivity. Higher scores indicate more severe attention-deficit and hyperactivity symptoms, and the cutoff score for the ADHD criteria is a total score of 19. In this study, the parents completed the Korean version of the ADHD Rating Scale-IV (K-ARS-IV) to measure the ADHD symptoms of the participants during the past 3 months. The K-ARS has been shown to have high reliability and validity to discriminate and identify children with ADHD (So et al., 2002; Kim et al., 2003).

#### 2.2.2 The advanced test of attention

The ATA is a computerized continuous performance test used to assess attention and inhibitory control (Cho et al., 2000). The test consists of visual and auditory subtests, each lasting 15 min. The participants were instructed to press the button (response) for the target stimuli as fast as possible and ignore the non-target stimuli (inhibit). Three nonverbal stimuli were presented for each subtest, all of which were non-target stimuli except for one target stimulus (Cho et al., 2000). In the visual test, an image with a triangle inside a square was the target stimulus, and the non-target stimuli were circles and squares drawn within a square. The target stimulus of an auditory test to which the participant should respond was three consecutive beep sounds; if two or four consecutive beeps were presented, the participants were not required to press the button. Each subtest was implemented after the practice trial according to the researcher’s instructions. The test yielded four main indicators: (1) omission error to measure the symptoms of inattention; (2) commission error to measure impulsivity and disinhibition; (3) mean response time (RT) for correct responses to measure the speed at which target stimuli were processed; and (4) response time variability (RTV) to measure the consistency of attention. All scores were translated into T-scores, adjusted based on normative distribution according to age and sex, with an average score of 50 and a standard deviation of 15.

#### 2.2.3 Stroop Color-Word Test

The participants underwent fNIRS assessment during SCWT administration. The SCWT is a frequently used neuropsychological test for evaluating cognitive control and entails multiple cognitive processes, including selective attention, response inhibition, interference control, and speeded response (Buck et al., 2008). The test consisted of three subtests (word, color, and color-word), each lasting 45 s. First, during the word subtest, participants were instructed to look at black-printed words and read the letters of the words as quickly as possible within the time limit. During the color test, the participants were instructed to read three types of color patches as quickly as possible. Finally, in the color-word subtest, the letters and colors of the words were presented incongruently, and the participants were instructed to say colors instead of letters. When the target (color) and obstruction (letter) stimuli were presented simultaneously, the ability to sustain attention was measured by inhibiting the interference of the obstruction stimuli and selecting only the target stimuli.

**Figure 1:**
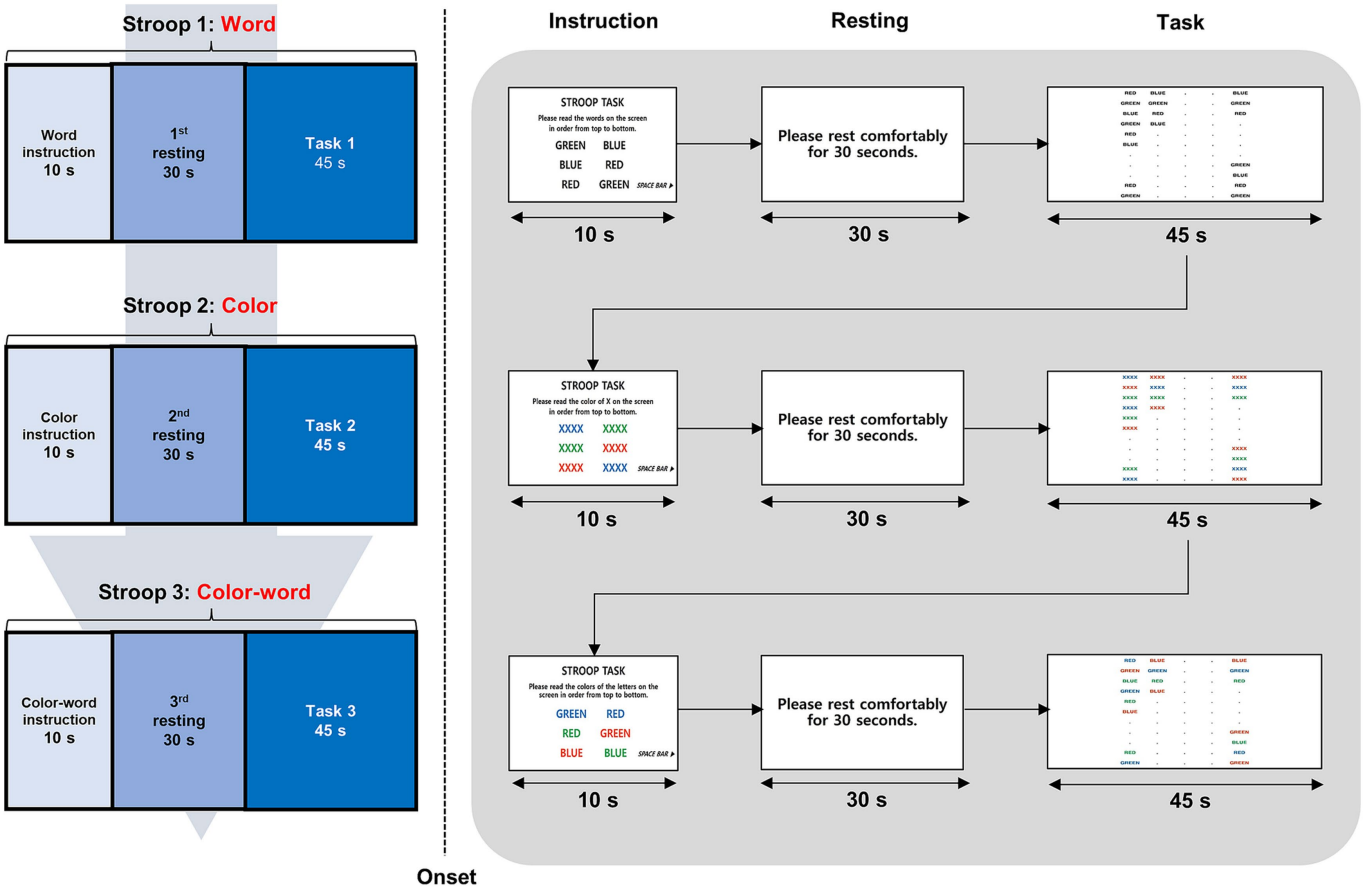
Task structure and experimental paradigm during fNIRS measurement. This figure illustrates the experimental paradigm, where participants performed three SCWT conditions—(1) Word, (2) Color, and (3) Color-Word—while wearing an fNIRS device. Each task block began with a 10 s instruction period, followed by a 30 s resting period, and ended with a 45 s cognitive task. fNIRS continuously measured changes in HbO and HbR in the prefrontal cortex, enabling the assessment of hemodynamic responses related to cognitive control and executive function. fNIRS, functional near-infrared spectroscopy; SWCT, Stroop Word Color Test; HbO, oxygenated hemoglobin; HbR, deoxygenated hemoglobin.

In this study, the Korean version of the standardized SCWT was implemented using E-Prime 3.0 software (Psychology Software Tools, Pittsburgh, PA, United States). For each subtest condition, after being guided by each test instructions and 30s of resting state, the participants proceeded to perform the task while watching the stimuli on the screen. The stimuli were presented in five rows with 20 words in each row. The raw score was calculated by counting the total number of correct answers for each test, and the interference score was calculated as the difference between the color and color-word scores. All scores were converted to T-scores based on the normative distribution according to age and sex. The protocol for SCWT administration during fNIRS assessment is shown in Figure 1.

### 2.3 fNIRS data acquisition and pre-processing

To quantify the amount of oxygen in the blood, we used a NIRSIT Lite Kids machine (OBELAB, Seoul, Republic of Korea), which is an fNIRS instrument with five dual-wavelength (780/850 nm) light-emitting diode light sources and 7 detectors spaced 2.5 cm apart, comprising 15 regular (2.5 cm) channels covering the prefrontal cortex. The device used a fixed optode configuration with predetermined channel locations. Channel positions were aligned with Montreal Neurological Institute coordinates and anatomical labels provided by the manufacturer. Based on this information, the channels were grouped into broader anatomical regions of interest, including the orbitofrontal, dorsolateral, and rostrolateral prefrontal cortices, reflecting the spatial organization of the device’s default configuration.

**Figure 2:**
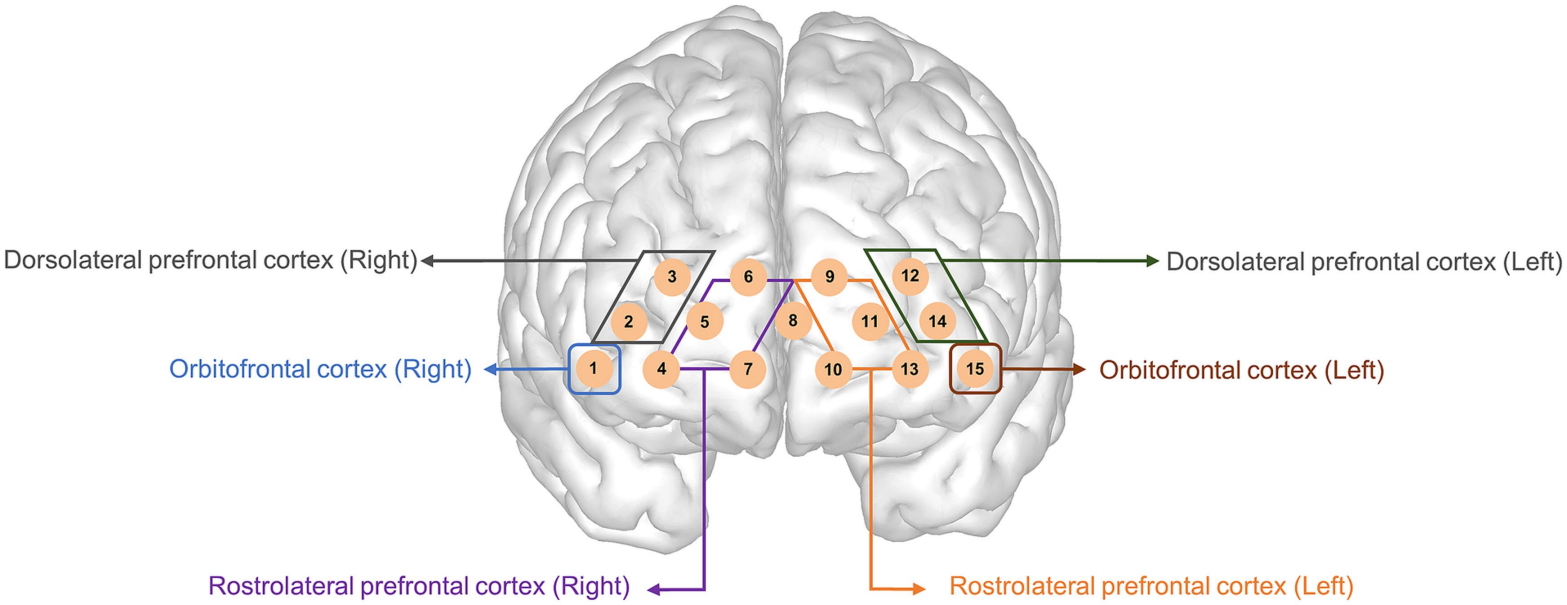
Topographical distribution of the 15 fNIRS recording channels within the prefrontal cortex. This figure illustrates the placement of the 15 fNIRS channels over the prefrontal cortex, covering the orbitofrontal, dorsolateral prefrontal, and rostrolateral prefrontal cortices. Channels on the left and right hemispheres were symmetrically positioned to measure oxygenated and deoxygenated hemoglobin changes. fNIRS, functional near-infrared spectroscopy.

The 15 channels are spaced 2.5 cm apart from each other, with 8 channels on the right side of the orbitofrontal cortex (channel 1), the dorsolateral prefrontal cortex (channels 2, 3), and the rostrolateral prefrontal cortex (channels 4, 5, 6, 7, 8), respectively, and 7 channels on the left side of the orbitofrontal cortex (channel 15), the dorsolateral prefrontal cortex (channels 12, 14), and the rostrolateral prefrontal cortex (channels 8, 9, 10, 11, 13). Channel 8 was excluded from the analysis because it belongs to both sides of the left and right rostrolateral prefrontal cortices. The alignment of the 15 channels is shown in Figure 2. If more than three of the 15 channels were disconnected, the subjects were excluded from the analysis. The optical-signal variation in each channel was sampled at a frequency of 8.138 Hz. The threshold of the signal-to-noise ratio criterion for determining poor quality, such as the slow drift of physiological noise and environmental noise, was 30 dB, which was used to qualify the noise of the detected channels after bandpass filtering from 0.005 to 0.1 Hz. However, the software does not support advanced denoising techniques, such as principal component analysis, independent component analysis, or short-separation regression for the removal of global physiological noise. The obtained optical intensity signals were converted into an oxygenated hemoglobin (HbO) concentration change time series using the modified Beer–Lambert law. The average signal for each channel during the last 10 s of rest and 30 s of each task was used in the analysis to compare regional neural activity between the groups. We focused on HbO rather than hemodynamic of deoxygenated hemoglobin (HbR) because HbR is 1–2 s slower than changes in HbO and may not reflect an accurate signal (Jasdzewski et al., 2003).

### 2.4 fNIRS: calculation of network parameters

Network-weighted edges were defined to obtain a functional network of the fNIRS data (Wang et al., 2020). Group-averaged connectivity data were obtained by averaging the time series of each resting and task block and then transforming the average of each participant’s Fisher-transformed value back to the Pearson’s correlation matrix. We conducted one-sample *t*-tests to compare the correlation values of each channel pair with a 0. The 15 network nodes had the same anatomical locations as the 15 channels and encompassed the orbitofrontal and rostrolateral prefrontal cortices. Global and local network metrics were analyzed using GRETNA software^1^ (Wang et al., 2020). The evaluated metrics included: (1) Global efficiency (Ge), which indicates the global efficiency of the parallel transfer of information in the network (Latora and Marchiori, 2001). Ge reflects network integration (Fischi-Gomez et al., 2016); (2) the nodal clustering coefficient (NC_p_), which indicates whether each node is interconnected with its neighbors (Onnela et al., 2005). The NC_p_ reflects network segregation (Lin et al., 2014; Fischi-Gomez et al., 2016; Jang et al., 2022); and (3) the weighted degree centrality (DC_w_), which is the sum of all weighted connections to a given node (Rubinov and Sporns, 2010).

### 2.5 MRI acquisition and analysis

Individuals with ADHD were scanned using a 3 T MRI (Philips, Achieva, 16-channel phase-array head coil, Best, Netherlands). In the T1-weighted images, the single-shot three-dimensional echo-planar images were obtained using these parameters: slice thickness = 1 mm, voxel sizes = 0.9 mm^2^, field of view = 224 mm^2^, repetition time = 8.3 ms, echo time = 4.6 ms, inverse time = 1 ms, and flip angle = 8°. The slice orientation was axially parallel to the anterior–posterior commissure line. A total of 150 slices contained the entire hemisphere and brainstem.

Automated reconstruction and segmentation of individual high resolution T1 weighted MRI volumes were performed using FreeSurfer version 7.1.1.^2^ The processing pipeline comprises motion correction, automated Talairach transformation, signal intensity normalization, removal of nonbrain tissue, automated correction for topological defects, subcortical segmentation, and cortical parcellation (Collins et al., 1994; Dale et al., 1999; Fischl et al., 1999; Fischl et al., 2002). The imaging process has been described in detail in previous studies (Fischl et al., 2002; Fischl et al., 2004). Regions based on the Desikan-Killiany atlas were segmented for volumetric analysis, with 34 left- and 34 right-hemispheric regions. To adjust for individual differences in the skull size, we corrected the absolute volume to the relative volume. The relative volume index was calculated as follows:


$$\text{Relative volume}\;(\%)=\frac{\text{Region of interest}}{\text{Intracranial volume}}\times 100$$

Thalamus segmentation was performed by using a sequence-adaptive Bayesian algorithm based on a probabilistic atlas (Iglesias et al., 2018). The segmented thalamus exhibited a volume of 25 regions in both the right and left thalami. These brain regional volumes were extracted with “asegstats2table” command. The segmentation outputs were visually inspected by two independent researchers (H. A. and Y. H.). Higher-order thalamic lesions can synchronize the activity of neural networks underlying various cognitive functions, including arousal, memory processing, attention, and reward-based behaviors (Jones, 2009; Saalmann, 2014). To evaluate the regions of interest of the thalamus related to the projection pathway to the frontal lobe, we selected the medial group (mediodorsal lateral parvocellular, mediodorsal medial magnocellular, reuniens, and paratenial) and the intralaminar group (central medial, centromedian, center-lateral, paracentral, and parafascicular).

The difference between the left and right hemispheres of each gross and subsegmentation of the thalamic region is represented by the asymmetry index. The asymmetry index was calculated using the following formula:


$$\text{Asymmetry index}=\frac{L-R}{\left(L+R\right)}$$

Where L and R denote the values of the left and right sides of the hemisphere, respectively.

### 2.6 Statistical analysis

All statistical analyses were performed using SPSS version 28.0 (IBM Corp., Armonk, NY, United States) and R version 4.3.1 (R Foundation for Statistical Computing, Vienna, Austria). Data visualization was conducted using GraphPad Prism version 9.0 (GraphPad Software, Inc., San Diego, CA, United States). Normality of continuous variables was tested using the Kolmogorov–Smirnov test. Based on normality results, independent samples *t*-tests or Mann–Whitney U tests were applied as appropriate to compare demographic and clinical characteristics and mean HbO levels between medicated and unmedicated ADHD groups. Bonferroni-corrected *p*-values were calculated using the NIRSIT Lite Analysis Tool (OBELAB, Seoul, Republic of Korea).

Group differences in fNIRS network metrics (global efficiency, clustering coefficient, nodal clustering coefficient, and weighted degree centrality) were examined using analysis of covariance (ANCOVA), adjusting for age and sex. Correlations between network metrics and Stroop performance were analyzed using Pearson’s correlation. Interaction effects between the medication group and psychological assessment scores (e.g., ADHD-RS, ATA, SCWT) on HbO levels were also tested using ANCOVA, with age, sex, medication type, and handedness included as covariates. The model was specified as follows:


$$\begin{array}{l} \mathrm{HbO}=\beta 0+\beta ₁(\text{Group}i)+\beta ₂(\text{Clinical}i)+\beta ₃(\begin{array}{l} \text{Group}i\times \\ \text{Clinical}i \end{array})+\\ \beta ₄(\mathrm{Sex}i)+\mathrm{\beta i}(\mathrm{Age}i)+\mathrm{\beta i}(\text{MedicationType}i)+\\ \mathrm{\beta i}(\text{Handedness}i)+\mathrm{\varepsilon i} \end{array}$$

where Clinical_i_ refers to a standardized psychological assessment score (e.g., response time variability from the ATA, total ADHD-RS score, or interference score from the SCWT). The interaction term (*β*_3_) captured whether the relationship between psychological scores and HbO concentration differed by group. For significant interactions, simple effect analyses were conducted to examine group-specific associations.

To examine hemispheric lateralization patterns, independent samples *t*-tests were performed on asymmetry scores between the two groups. Group comparisons of thalamic asymmetry scores were conducted using general linear models adjusted for age and sex. Multiple comparison correction was not applied, as the asymmetry indices were predefined region-level summary measures rather than voxel-wise or high-dimensional data requiring control for family-wise error. Statistical significance was set at a two-tailed *p*-value of < 0.05.

## 3 Results

### 3.1 Demographic and clinical characteristics

The demographic and clinical characteristics of the study participants are shown in Table 1. A total of 45 children with ADHD (23 medicated and 22 unmedicated) were evaluated. The participant groups did not differ in terms of mean age, sex, full-scale IQ, ADHD-RS-IV inattention, or socioeconomic characteristics. The ADHD-RS-IV hyperactivity–impulsivity scores were significantly higher in the unmedicated ADHD group than in the medicated ADHD group (11.91 ± 5.08 vs. 8.83 ± 4.15, *p* = 0.04). The RTV score on the ATA visual task was also significantly higher in the unmedicated group compared to the medicated group (67.77 ± 17.39 vs. 57.30 ± 19.37, *p* = 0.03). There were no significant differences in the ATA auditory task and SCWT parameter scores. The CGI-S score was significantly lower in the medicated ADHD group compared to the unmedicated ADHD group (4.22 ± 0.4 vs. 4.62 ± 0.59, *p* = 0.02).

### 3.2 Comparison of hemodynamic differences between groups

**Figure 3:**
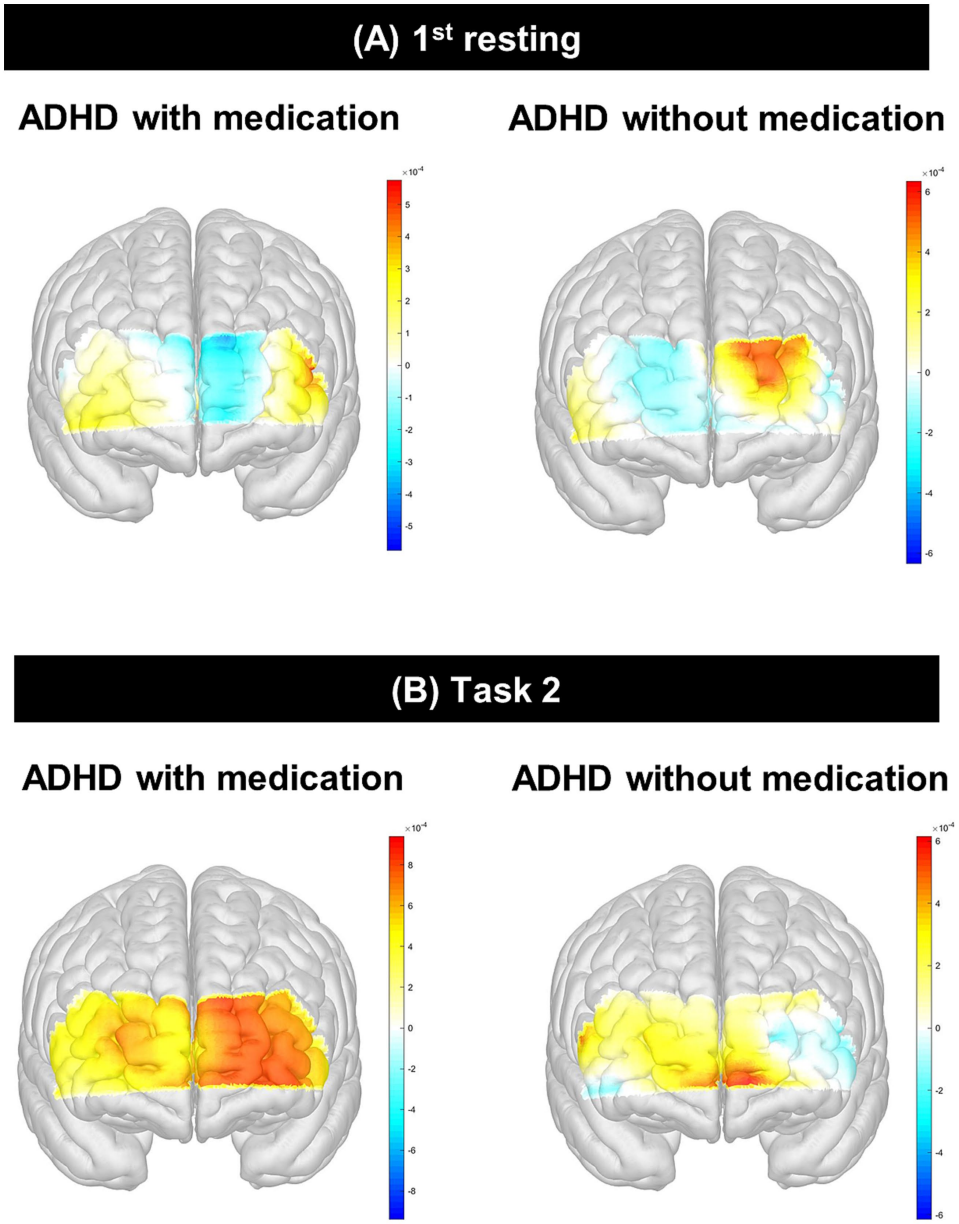
Interaction effect between psychological test and group using ANCOVA. **(A)** shows ATA visual commission error × group interaction in channel 9 during the 1st resting state; **(B)** shows ATA visual RTV × group interaction in channel 9 during the 1st resting state, with a significant effect (*p* = 0.02); **(C)** shows ARS inattention × group interaction in channel 13 during Task 2 (Color); **(D)** shows ATA visual commission error × group interaction in channel 13 during Task 2 (Color). ATA, advanced test of attention; HbO, hemoglobin oxygen; SD, standard deviation; RTV, response time variability; ARS, ADHD rating scale; ADHD, attention deficit hyperactivity disorder; ANCOVA, analysis of covariance. *p < 0.05.

The HbO of channel 9 (*p* = 0.01) in the 1st rest period and the HbO of channels 13 (*p* = 0.02) and 14 (*p* = 0.01) during task 2 (color) were significantly different after Bonferroni correction. The medicated ADHD group showed significantly lower HbO than the unmedicated ADHD group in channel 9 of 1st rest (−0.40 ± 0.13 vs. 0.61 ± 0.11). Contrastingly, the medicated ADHD group showed significantly higher HbO than those in the unmedicated group in channel 13 and 14 during task 2 (color test) (0.82 ± 0.10 vs. 0.06 ± 0.11, 0.59 ± 0.10 vs. − 0.19 ± 0.10, respectively). The differences in HbO levels between the two groups are shown in Table 2 and Figure 3 and HbR was found in Supplementary Table 1. However, there was no significant difference in hemispheric asymmetry score of HbO and HbR between groups (Supplementary Table 2).

**Table 2:** Differences in mean oxyhemoglobin measurements between participants with ADHD with and without medication.

Section	Channel	Medicated ADHD (*n* = 23)	Unmedicated ADHD (*n* = 22)	*p*	Section	Channel	Medicated ADHD (*n* = 23)	Unmedicated ADHD (*n* = 22)	*p*
1st rest	1	0.171 ± 0.11	0.346 ± 0.08	0.56	Task 1 (Word)	1	0.032 ± 0.09	−0.071 ± 0.10	0.73
	2	−0.061 ± 0.14	0.174 ± 0.12	0.55		2	0.444 ± 0.11	0.671 ± 0.18	0.62
	3	0.053 ± 0.10	0.021 ± 0.11	0.92		3	0.492 ± 0.11	0.144 ± 0.13	0.33
	4	0.297 ± 0.12	0.194 ± 0.13	0.78		4	0.173 ± 0.11	−0.144 ± 0.12	0.37
	5	0.121 ± 0.18	−0.177 ± 0.12	0.51		5	0.570 ± 0.17	0.191 ± 0.11	0.38
	6	−0.117 ± 0.12	−0.186 ± 0.17	0.86		6	0.184 ± 0.13	0.063 ± 0.10	0.73
	7	0.091 ± 0.11	−0.123 ± 0.10	0.49		7	0.332 ± 0.13	−0.144 ± 0.09	0.17
	8	−0.066 ± 0.08	−0.083 ± 0.14	0.96		8	0.147 ± 0.14	0.122 ± 0.13	0.95
	9	−0.400 ± 0.13	0.613 ± 0.11	0.01*		9	0.231 ± 0.21	−0.312 ± 0.18	0.36
	10	−0.259 ± 0.11	−0.227 ± 0.09	0.92		10	0.196 ± 0.17	0.359 ± 0.05	0.67
	11	0.104 ± 0.11	0.558 ± 0.17	0.30		11	0.358 ± 0.16	0.222 ± 0.16	0.78
	12	0.065 ± 0.18	0.049 ± 0.13	0.36		12	0.340 ± 0.11	−0.044 ± 0.17	0.37
	13	0.234 ± 0.17	0.005 ± 0.12	0.60		13	0.402 ± 0.10	−0.153 ± 0.13	0.12
	14	0.504 ± 0.13	−0.183 ± 0.14	0.09		14	0.265 ± 0.11	0.447 ± 0.11	0.58
	15	0.000 ± 0.12	0.146 ± 0.13	0.69		15	0.523 ± 0.19	0.316 ± 0.15	0.68
2nd rest	1	−0.112 ± 0.05	−0.051 ± 0.12	0.83	Task 2 (Color)	1	0.475 ± 0.08	−0.111 ± 0.11	0.05
	2	0.178 ± 0.16	−0.047 ± 0.13	0.15		2	0.462 ± 0.21	0.489 ± 0.20	0.97
	3	0.127 ± 0.09	0.035 ± 0.10	0.75		3	0.393 ± 0.08	0.108 ± 0.11	0.33
	4	0.070 ± 0.08	−0.317 ± 0.12	0.20		4	0.240 ± 0.08	−0.145 ± 0.10	0.18
	5	0.219 ± 0.13	−0.305 ± 0.12	0.17		5	0.730 ± 0.18	0.377 ± 0.19	0.52
	6	0.406 ± 0.11	−0.067 ± 0.11	0.17		6	0.547 ± 0.11	0.084 ± 0.10	0.14
	7	0.300 ± 0.16	−0.244 ± 0.09	0.08		7	0.571 ± 0.10	0.415 ± 0.17	0.71
	8	0.058 ± 0.06	−0.342 ± 0.09	0.08		8	0.418 ± 0.10	0.163 ± 0.15	0.51
	9	−0.143 ± 0.14	−0.117 ± 0.13	0.95		9	0.865 ± 0.21	0.153 ± 0.16	0.21
	10	−0.266 ± 0.15	−0.597 ± 0.11	0.40		10	0.825 ± 0.17	0.852 ± 0.24	0.97
	11	0.555 ± 0.18	−0.131 ± 0.16	0.18		11	0.727 ± 0.19	−0.260 ± 0.11	0.07
	12	0.003 ± 0.07	−0.013 ± 0.11	0.87		12	0.670 ± 0.10	0.194 ± 0.13	0.24
	13	0.048 ± 0.11	−0.804 ± 0.18	0.06		13	0.818 ± 0.10	0.057 ± 0.11	0.02*
	14	0.091 ± 0.15	−0.030 ± 0.07	0.73		14	0.592 ± 0.10	−0.188 ± 0.10	0.01*
	15	0.114 ± 0.10	0.094 ± 0.14	0.95		15	0.334 ± 0.13	−0.120 ± 0.21	0.38
3rd rest	1	0.195 ± 0.07	0.426 ± 0.11	0.40	Task 3 (Color-Word)	1	0.107 ± 0.12	0.129 ± 0.15	0.24
	2	0.898 ± 0.25	0.403 ± 0.20	0.47		2	0.582 ± 0.11	0.360 ± 0.20	0.97
	3	0.213 ± 0.11	0.206 ± 0.14	0.99		3	0.505 ± 0.10	0.447 ± 0.11	0.55
	4	0.410 ± 0.10	0.114 ± 0.13	0.39		4	0.481 ± 0.08	0.022 ± 0.09	0.44
	5	0.132 ± 0.16	0.385 ± 0.17	0.61		5	0.358 ± 0.08	0.200 ± 0.11	0.65
	6	−0.167 ± 0.28	0.161 ± 0.12	0.61		6	0.455 ± 0.13	0.289 ± 0.10	0.41
	7	−0.118 ± 0.17	0.413 ± 0.14	0.27		7	0.412 ± 0.11	−0.011 ± 0.11	0.82
	8	0.162 ± 0.27	0.162 ± 0.27	0.91		8	0.386 ± 0.15	0.065 ± 0.07	0.65
	9	−0.332 ± 0.33	0.195 ± 0.14	0.49		9	0.173 ± 0.30	0.184 ± 0.12	0.44
	10	−0.163 ± 0.25	0.526 ± 0.13	0.25		10	0.394 ± 0.18	0.333 ± 0.11	0.97
	11	−0.484 ± 0.46	0.379 ± 0.13	0.40		11	0.085 ± 0.15	0.058 ± 0.12	0.26
	12	−0.122 ± 0.21	0.510 ± 0.18	0.28		12	−0.597 ± 0.21	0.439 ± 0.09	0.44
	13	0.459 ± 0.21	0.469 ± 0.15	0.99		13	0.303 ± 0.10	0.265 ± 0.09	0.17
	14	0.505 ± 0.14	0.505 ± 0.13	1.00		14	0.202 ± 0.11	−0.117 ± 0.11	0.15
	15	0.542 ± 0.19	0.004 ± 0.18	0.37		15	0.501 ± 0.19	0.359 ± 0.11	0.57

Data are presented as mean ± SD (×10^−3^). ADHD, attention deficit hyperactivity disorder; SD, standard deviation. * Statistically significant at *p* < 0.05, based on Bonferroni correction for multiple comparisons calculated using the NIRSIT Lite Analysis Tool. *Bold values indicate statistically significant differences at *p* < 0.05.

### 3.3 Correlation between HbO and clinical scores in groups

In channel 9, the HbO values measured during the 1st resting period showed a significant positive correlation with RTV scores on the ATA visual task in the unmedicated ADHD group (*p* = 0.02; Supplementary Table 3), after adjusting for sex, age, handedness, and medication type (atomoxetine, methylphenidate, or both). We conducted a simple effect analysis to examine the interaction between group and RTV scores on HbO levels in channel 9. The results, summarized in Supplementary Table 4, showed a negative slope in the unmedicated group (*β* = −0.021 × 10^−3^, *p* = 0.11), while the medicated group showed a positive slope (*β* = 0.025 × 10^−3^, *p* = 0.10), although neither effect was statistically significant. In channel 13, the HbO values of task 2 were negatively correlated with the hyperactivity-impulsivity scores and total ARS scores in the unmedicated ADHD group (Supplementary Table 5), while they were positively correlated with SCWT word scores in the medicated ADHD group after adjusting for sex, age, handedness, and medication type (atomoxetine, methylphenidate, or both).

**Figure 4:**
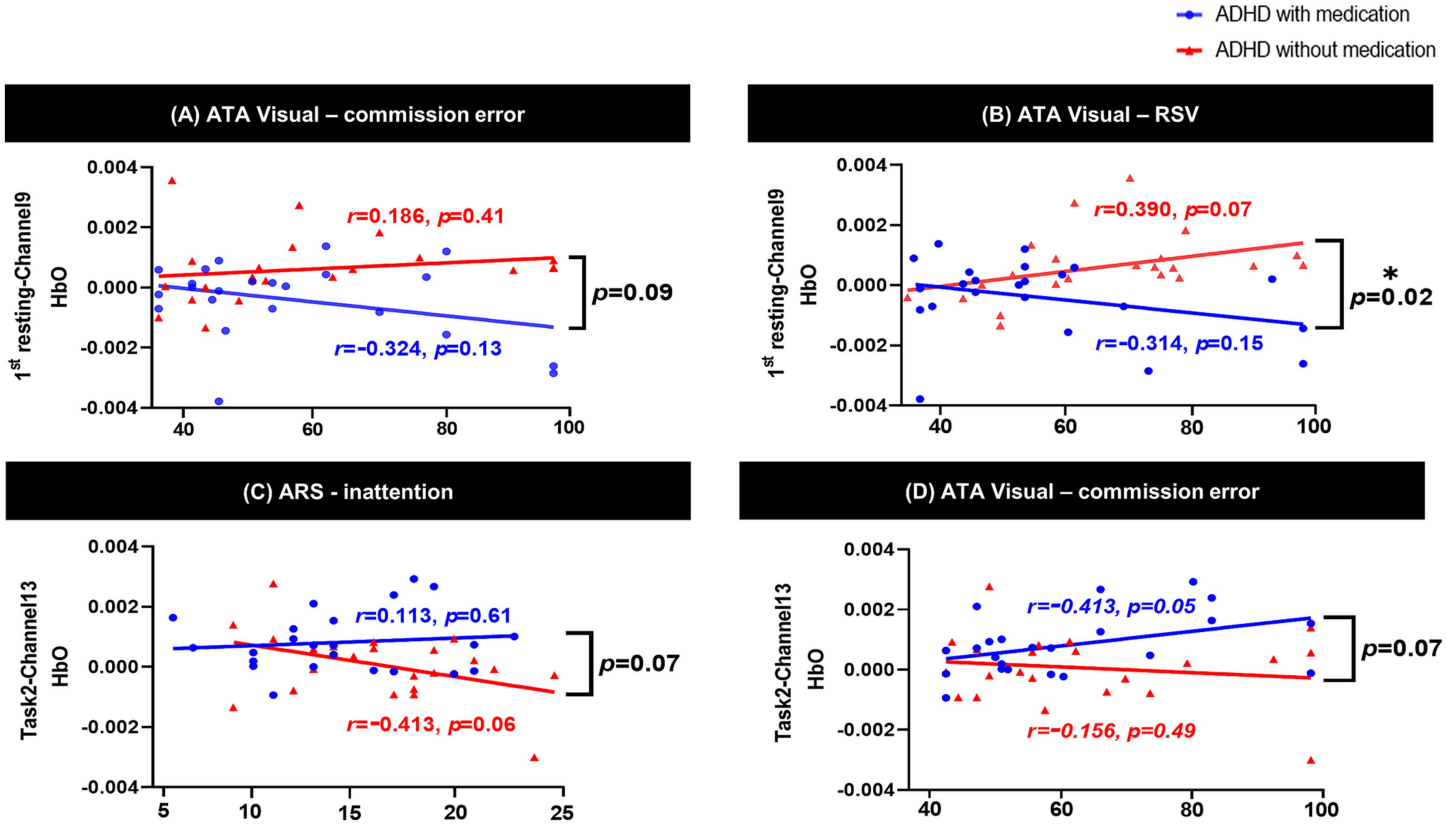
Differences of HbO between participants with ADHD with and without. This figure presents the comparative analysis of HbO levels measured using fNIRS in children with ADHD who were either medicated or unmedicated. **(A)** shows group differences in HbO during the 1st resting state, and **(B)** shows group differences during Task 2 (Color). The unmedicated ADHD group exhibited significantly lower activation in the left rostrolateral prefrontal cortex (channel 9, *p*=0.01; channel 13, *p*=0.02) and dorsolateral prefrontal cortex (channel 14, *p*=0.01) during the SCWT, while the medicated group showed increased HbO responses in these regions. HbO, hemoglobin oxygen; ADHD, attention deficit hyperactivity disorder; fNIRS, functional near-infrared spectroscopy; SWCT, Stroop Word Color Test.

In the interaction analyses, there was a significant interaction effect between RTV scores of the ATA visual task and the medicated ADHD group in channel 9 of the 1st rest period (*interaction p* = 0.02). In channel 9, the correlation between RTV scores and HbO during the 1st resting period showed opposite directions according to medication in the two groups; although not significant, there was a negative correlation in the medicated ADHD group and a positive correlation in the unmedicated ADHD group. There was a similar tendency in ATA visual task commission error [*interaction p* = 0.09 at channel 9 during 1st rest, *interaction p* = 0.07 at channel 13 during task 2 (color)] and ARS inattention scores [*interaction p* = 0.07 at channel 13 during task 2 (color)]. The interaction effects between the clinical scores and medication are shown in Figure 4.

### 3.4 Group comparison of fNIRS network metrics

To examine whether pharmacological treatment is associated with alterations in brain network organization, we compared global and local network metrics between the medicated and unmedicated groups. We found no significant differences between groups across all network metrics during both resting-state and task conditions after adjusting for age and sex (Supplementary Table 6).

### 3.5 Comparison of hemispheric asymmetry of gross volume between groups

**Figure 5:**
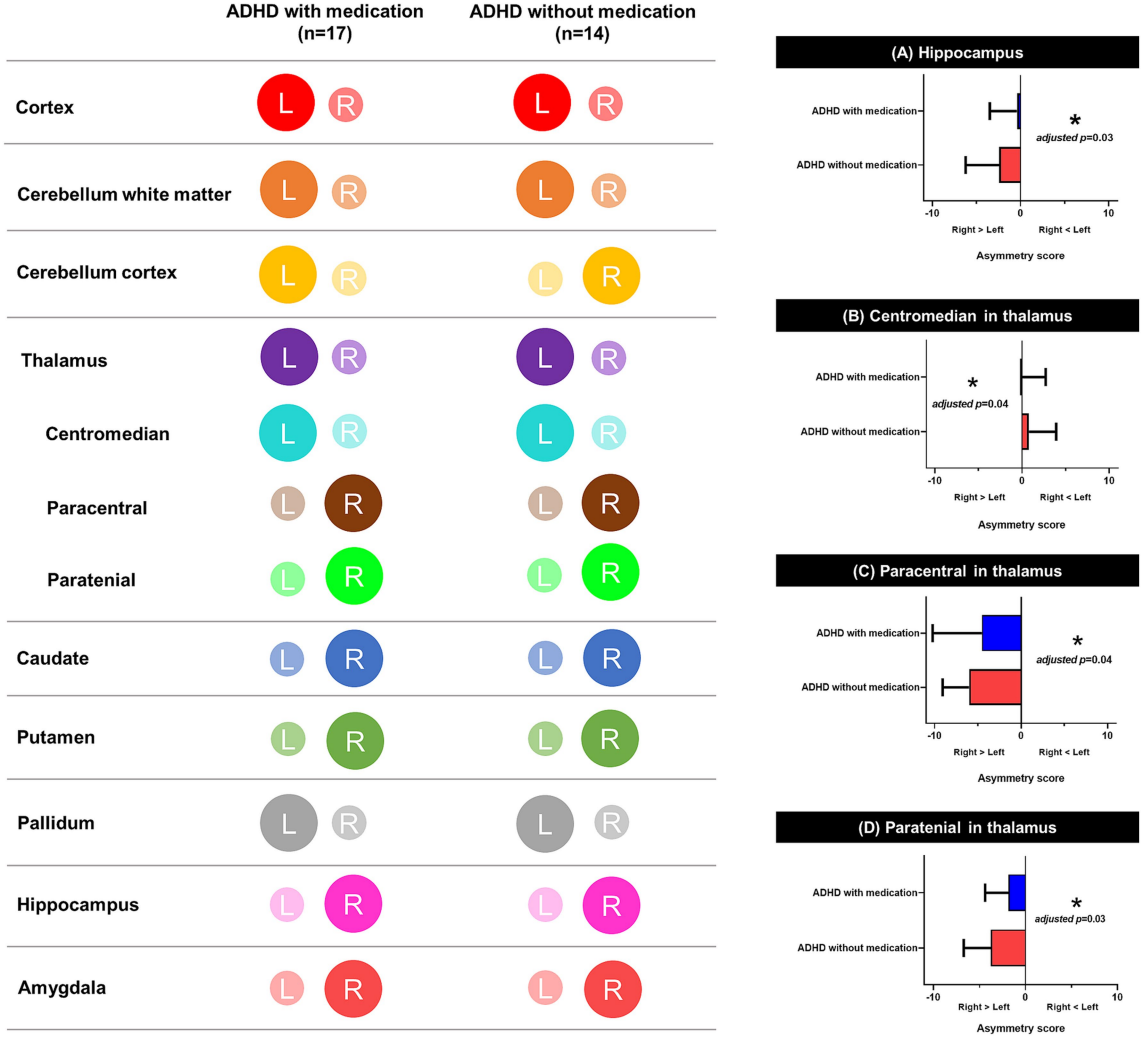
Schematic representation of lateralization on brain. This figure illustrates lateralization patterns across brain regions in children with ADHD, with and without medication. The color-coded maps on the left indicate hemispheric asymmetry in each region, and the bar graphs on the right show significant group differences in asymmetry scores. **(A)** Hippocampus, **(B)** Centromedian nucleus of the thalamus, **(C)** Paracentral nucleus of the thalamus, and **(D)** Paratenial nucleus of the thalamus all showed significant differences in asymmetry scores between groups. Asymmetry scores were calculated based on the absolute volumes adjusted for sex, age, handedness, and total volume excluding ventricles. *Adjusted *p* < 0.05. L, left hemisphere; R, right hemisphere; ADHD, attention deficit hyperactivity disorder.

There was no significant difference in the structural brain volume (Supplementary Table 7) between the medicated and unmedicated ADHD groups and among three groups categorized by type of medication (methylphenidate only, atomoxetine only, and both) (Supplementary Table 8). Figure 5 and Supplementary Table 9 show the asymmetric patterns in the volume of the cortex, white matter, cerebellum, and subcortical regions, including the hippocampus and thalamus. There was a similar pattern of lateralization between groups, as the medicated ADHD group tended to be lateralized to the left hemisphere and the unmedicated ADHD group tended to be lateralized to the right hemisphere. There were significant differences in the (a) hippocampus, (b) centomedian, (c) paracentral, and (d) paratenial of the thalamus after adjusting sex, age, handedness, and total brain volume excluded ventricle or total thalamus volume (adjusted *p* = 0.03, 0.04, 0.04, and 0.03, respectively) asymmetry scores between the medicated and unmedicated ADHD groups. Children with ADHD who were not taking medication showed more rightward asymmetry in the volumes of the thalamus and hippocampus than those with ADHD who were taking medication.

## 4 Discussion

To our knowledge, this is the first multimodal fNIRS-MRI study to compare the effect of medication during a cognitive task and the resting state, elucidating the neuropharmacological substrate based on thalamic asymmetry in children with ADHD. We observed differential brain activity, especially in the left prefrontal cortex during the SCWT, and reduced hemispheric asymmetry in thalamic volume in medicated compared to unmedicated children with ADHD. More specifically, we found reduced hemodynamic activity during the resting state, but increased HbO during the SCWT following pharmacotherapy. The difference in brain activity during the SCWT tasks according to medication was especially prominent in the left prefrontal cortex. Moreover, we found significantly reduced thalamic asymmetry in children with ADHD treated with medication, suggesting a pathoplastic role of neural substrates in the medication effect.

Most studies revealed increased HbO activation in the prefrontal cortex following medication and a higher frequency of right lateralization. Specifically, HbO activation increases after medication intake in the right dorsolateral and ventrolateral prefrontal cortices (Monden et al., 2012b). In other studies, bilateral increases in oxygenation due to medication (Akira, 2015; Ota et al., 2015) and left-lateralized treatment effects have been examined due to pharmacological effects during neural assessments (Sanefuji et al., 2014; Nakanishi et al., 2017). Another review reported inconsistent results among studies investigating the pharmacological effects of MPH and ATM (Grazioli et al., 2019). Some previous studies were limited to boys who did not receive psychotropic medication (Jourdan Moser et al., 2009; Tsujimoto et al., 2013) whereas others compared children with ADHD to healthy controls (Suzuki et al., 2017). Moreover, few studies have differentiated the neural substrates associated with brain activation using fNIRS in children with ADHD receiving medication compared to those not receiving medication. It is also noteworthy that the heterogeneity of the patient group, including both naïve and non-naïve children with ADHD, and the diversity of neurocognitive tasks, could be partly attributed to the inconsistent results (Grazioli et al., 2019). In our study, we found that prefrontal activity in medicated children with ADHD increased during cognitive tasks. This result is in line with previous findings that brain activation increases during the SCWT after taking medication (Nakanishi et al., 2017; Grazioli et al., 2019; Hou et al., 2022) indicating the restoration of brain activation with medication. As previous studies have used different tasks, different types of medication, and different statistical methods to investigate medication effects and have been small in sample size, more well-designed studies with larger sample sizes are needed.

A recent systematic review (Hou et al., 2022) showed that brain regions associated with inhibitory control and working memory are widely distributed in the bilateral prefrontal cortices of healthy people. The left prefrontal region is implicated in inhibitory functions and plays an important role in modulating ADHD symptoms (Konishi et al., 2005). The rostrolateral prefrontal cortex has a large number of dendritic spines per cell (Dumontheil et al., 2008) and is associated with higher-order cognitive functions (Boschin et al., 2015). Our results are consistent with those of Miao et al., in which children with ADHD exhibited consistent hypoactivity in the left rostrolateral prefrontal cortex during inhibition tasks (Miao et al., 2017). The left rostrolateral prefrontal cortex plays a role in controlling attentional reallocation (Pollmann et al., 2000), which is impaired in patients with ADHD. To investigate the function of the prefrontal cortex, we used a cognitive task, the SCWT, which is one of the best-known psychological experiments requiring high-level cognitive functions of response selection and response inhibition (Mutter et al., 2005). Using the SCWT, we identified that activation of the left rostrolateral prefrontal cortex measured by fNIRS could serve as an objective neural biomarker for children with ADHD.

The significant difference in brain activation in the left prefrontal cortex in the present fNIRS study is noteworthy, and in line with evidence related to the symptoms of the disease in children with ADHD. Notably, we found an interaction effect of brain activity in the left-brain channels between the two groups, suggesting inefficient hemodynamic processing of cognitive function in unmedicated patients with ADHD. The unmedicated ADHD group showed decreased HbO during cognitive tasks according to symptom severity, whereas they exhibited increased HbO in the resting state according to symptom severity. This interesting result regarding the aberrant distribution of hemodynamic changes when entering the cognitive workload extends previous results that showed reduced activation of the prefrontal cortex at fNIRS in children with ADHD beyond resting-state brain activity (Ishii et al., 2017; Kaga et al., 2020). As the difficulty of the task increases, children with ADHD and impaired cognitive function experience imbalances in effectively distributing hemodynamic energy (Matsuura et al., 2014). Additionally, because children with ADHD are debilitated in their ability to switch tasks, they show a specific deficit regarding the flexible suppression of different rules according to instructions (Cepeda et al., 2000). However, ADHD medication modulates prefrontal hemodynamic responses, potentially enhancing cognitive control and executive functioning. Consistent with previous findings that methylphenidate can enhance inhibitory control associated with attention switching, stimulant medication may modulate executive function in children with ADHD. Therefore, we conclude that alterations in hemodynamic maintenance reflect the effects of medication in the human brain when confronted with changing cognitive demands to achieve an optimal distribution of attention. Although group differences in hemodynamic activation were observed, no significant group differences were identified in functional connectivity metrics. This dissociation between local activation and preserved network organization suggests that while the magnitude of regional brain activity may vary by treatment status, the large-scale network structure remains stable. This preserved connectivity could serve as a compensatory framework that supports cognitive performance despite localized dysfunction (Deng et al., 2021; Fan et al., 2025).

However, we found a significant difference in asymmetry in the hippocampus and several thalamic volumes between medicated and unmedicated patients with ADHD on sMRI, suggesting a possible mechanism involved in the different cortico-subcortical connectivities. A previous study suggested that the volume of the prefrontal cortex in patients with ADHD was smaller, while the volume of the right subcortical region was greater, compared to typically developing children (He et al., 2022). The prefrontal cortex receives inputs via the thalamus from other cortical regions and subcortical structures, such as the hippocampus and amygdala, selecting relevant information and ignoring distracting stimuli. An increasing number of studies have shown that the thalamus is a key anatomical structure underlying normal attentional and cognitive control mechanisms in ADHD (Rubia et al., 2000; Hale et al., 2015). The thalamus helps suppress inappropriate responses and maintain cognitive flexibility in important tasks. Ivanov et al. reported that patients with ADHD receiving medication had larger thalamic volumes than those who were not receiving medication, compensating for insufficient prefrontal function (Batty et al., 2015). Particularly, the intralaminar and medial nuclei of the thalamus have been implicated in synchronizing cortical neurons for effective information transmission during cognitive processing (Saalmann, 2014). Additionally, the hippocampus is involved in the neural network of learning and working memory, which is a key defect in patients with ADHD (Squire and Wixted, 2011; Ezzati et al., 2016).

Although brain asymmetry is a unique feature of the human brain, aberrations in its asymmetric nature may represent plausible clues regarding the effect of ADHD on brain asymmetry, such as abnormal brain development and maturation processes. Recent studies using NIRS have reported functional asymmetry in hemodynamic activation with regard to hemispheric differences as potential biomarkers in children with ADHD (Wang et al., 2020). However, they did not compare the structural asymmetry of the brain volume between medicated and unmedicated children with ADHD. Our findings show plausible evidence of abnormal hemispheric asymmetry as the neural basis of the core deficit associated with ADHD, in which unmedicated children with exhibited increased asymmetry in the brain volume of the thalamus and hippocampus compared to medicated children with ADHD. Altered hemispheric asymmetry in the hippocampus and thalamus may contribute to imbalanced attention control under disease conditions, leading to altered cortico-subcortical connectivity with disrupted projections from the subcortex to the frontal cortex. Consistent with our findings, Douglas et al. revealed morphometric asymmetry of the hippocampus and thalamus in patients with ADHD compared to TD (Douglas et al., 2018). Moreover, the degree of hemispheric asymmetry involving the prefrontal and subcortical regions is higher in children with more severe ADHD symptoms, indicating a neuroplastic role for disrupted neural mechanisms in the inhibition of cognitive interference (He et al., 2022).

This study has several limitations. First, the medicated group may have included individuals with suboptimal treatment response. The study enrolled patients with ADHD into two groups, with all participants having a CGI-S score exceeding 4. This inclusion criterion raises the possibility that the medicated group included individuals who had not adequately responded to treatment, particularly in terms of psychobehavioral phenotypes. These factors may have confounded the neuroimaging findings. Second, the cross-sectional observational design did not control for treatment-related variables such as medication dosage, duration, or timing, which may have affected the observed neural responses. Future studies should rigorously account for these factors to better clarify causal relationships. Third, fNIRS measurements were limited to the prefrontal cortex due to the constraints of the 15-channel pediatric system, preventing the assessment of parietal or temporal regions involved in ADHD. Broader cortical coverage using multichannel fNIRS or fMRI is recommended for future research. Fourth, baseline ADHD-RS scores differed between groups, likely reflecting medication effects at the time of measurement rather than pre-treatment severity. Nevertheless, this group difference may introduce selection bias. Finally, all ADHD subtypes were included due to limited sample size, potentially contributing to variability in behavioral and neuroimaging patterns.

## 5 Conclusion

In summary, we examined the effect of medication on hemodynamic activity and functional network connectivity in the prefrontal area using fNIRS and on the hemispheric asymmetry of structural brain volume with MRI in children with ADHD. Our findings suggest that left rostrolateral prefrontal activation and reduced thalamic asymmetry are important for inhibitory control, and that the activity of this region is restored by ADHD medication.

## Data Availability

The raw data supporting the conclusions of this article will be made available by the authors, without undue reservation.

## Footnotes

^1^ http://www.nitrc.org/projects/gretna/

^2^ https://surfer.nmr.mgh.harvard.edu/
