# Supplementary material for: Effect of medication on the rostrolateral prefrontal oxygenation and thalamic volume asymmetry in youths with ADHD
Source: Front Integr Neurosci. 2025 May 21;19:1591465. doi: 10.3389/fnint.2025.1591465 (PMC12135343; doi:10.3389/fnint.2025.1591465)
Supplement: Supplementary file 1 [file Table_1.docx]

Supplementary Material

Effect of medication on the rostrolateral prefrontal oxygenation and thalamic volume asymmetry in ADHD youth

Hyuna Kim^1,^ ^†^, Da Hyun Kang^2, †^, Yong Hun Jang^1^, Ja-Hye Ahn^3^, Sojin Won^4^, Hyun Ju Lee ^3, ‡, *^, Johanna Inhyang Kim^2,4,5,^ ^‡, *^

^1^ Department of Translational Medicine, Hanyang University Graduate School of Biomedical Science and Engineering, Seoul, Republic of Korea

^2^ Institute of Mental Health, Hanyang University, Seoul, Republic of Korea

^3^ Department of Pediatrics, Hanyang University Hospital, Hanyang University College of Medicine, Seoul, Republic of Korea

^4^ Hanyang Inclusive Clinic for Developmental Disorders, Seoul, Republic of Korea

^5^ Department of Psychiatry, Hanyang University Medical Center, Seoul, Republic of Korea

† These authors contributed equally to this work and share the first authorship.

‡ These authors contributed equally to this work and share the corresponding authorship.

*** Correspondence:** Corresponding Authors: 1) Hyun Ju Lee, blesslee77@hanmail.net 2) Johanna Inhyang Kim, iambabyvox@hanmail.net

# Supplementary Tables

**Supplementary Table 1.** Differences in mean deoxyhemoglobin measurements between medicated and unmedicated ADHD children

| **section** | **channel** | **Medicated ADHD**  **(n=23)** | **Unmedicated ADHD**  **(n=22)** | ***p*** | **section** | **channel** | **Medicated ADHD**  **(n=23)** | **Unmedicated ADHD**  **(n=22)** | ***p*** |
| --- | --- | --- | --- | --- | --- | --- | --- | --- | --- |
| 1^st^  rest | 1 | 0.16±1.10 | 0.30±0.84 | 0.63 | Task1  (Word) | 1 | 0.52±0.78 | -0.15±1.09 | **0.02*** |
|  | 2 | -0.10±1.39 | -0.01±1.31 | 0.84 |  | 2 | 0.53±2.10 | 0.53±2.01 | 1.00 |
|  | 3 | 0.03±1.01 | -0.09±1.09 | 0.71 |  | 3 | 0.33±0.82 | 0.17±1.08 | 0.58 |
|  | 4 | 0.29±1.17 | 0.17±1.29 | 0.58 |  | 4 | 0.25±0.83 | -0.20±0.97 | 0.10 |
|  | 5 | 0.14±1.79 | -0.09±1.32 | 0.63 |  | 5 | 0.77±1.74 | 0.14±1.50 | 0.20 |
|  | 6 | -0.13±0.87 | -0.18±1.65 | 0.88 |  | 6 | 0.50±1.12 | 0.10±0.91 | 0.21 |
|  | 7 | 0.09±1.07 | -0.05±1.20 | 0.68 |  | 7 | 0.58±1.00 | 0.11±0.94 | 0.11 |
|  | 8 | -0.09±0.83 | -0.05±1.39 | 0.92 |  | 8 | 0.34±0.92 | 0.08±1.11 | 0.42 |
|  | 9 | -0.41±1.30 | 0.57±1.10 | **0.01*** |  | 9 | 0.85±2.11 | 0.28±1.54 | 0.31 |
|  | 10 | -0.31±1.15 | 0.00±1.18 | 0.40 |  | 10 | 0.85±1.70 | 0.18±0.94 | 0.13 |
|  | 11 | 0.15±1.11 | 0.60±1.84 | 0.36 |  | 11 | 0.49±1.96 | -0.10±1.20 | 0.25 |
|  | 12 | 0.17±1.68 | 0.42±1.32 | 0.60 |  | 12 | 0.52±1.28 | 0.13±1.31 | 0.32 |
|  | 13 | 0.23±1.66 | -0.01±1.30 | 0.59 |  | 13 | 0.82±1.01 | 0.04±1.14 | **0.02*** |
|  | 14 | 0.50±1.25 | -0.22±1.37 | 0.07 |  | 14 | 0.63±0.94 | -0.15±0.96 | **0.01*** |
|  | 15 | 0.01±1.17 | 0.04±1.35 | 0.94 |  | 15 | -0.15±0.96 | 0.34±1.28 | 0.44 |
| 2^nd^ rest | 1 | 0.11±1.02 | -0.05±0.94 | 0.58 | Task2  (Color) | 1 | 0.20±0.67 | 0.37±1.13 | 0.56 |
|  | 2 | 0.53±1.15 | 0.83±1.70 | 0.50 |  | 2 | 1.03±2.39 | 0.06±2.10 | 0.16 |
|  | 3 | 0.52±1.12 | 0.23±1.11 | 0.39 |  | 3 | 0.30±1.09 | -0.00±1.28 | 0.40 |
|  | 4 | 0.29±1.26 | -0.20±1.06 | 0.16 |  | 4 | 0.50±0.94 | 0.09±1.28 | 0.23 |
|  | 5 | 0.66±1.70 | 0.31±0.96 | 0.40 |  | 5 | 0.20±1.54 | 0.27±1.64 | 0.88 |
|  | 6 | 0.22±1.34 | 0.16±0.96 | 0.86 |  | 6 | -0.10±2.77 | 0.11±1.20 | 0.75 |
|  | 7 | 0.42±1.39 | -0.12±0.75 | 0.11 |  | 7 | 0.04±1.85 | 0.20±1.36 | 0.75 |
|  | 8 | 0.20±1.46 | -0.02±1.18 | 0.59 |  | 8 | 0.23±2.71 | 0.02±0.82 | 0.73 |
|  | 9 | 0.29±2.15 | -0.34±1.83 | 0.30 |  | 9 | -0.29±3.30 | 0.21±1.30 | 0.51 |
|  | 10 | 0.28±1.80 | -0.02±0.86 | 0.52 |  | 10 | -0.04±2.57 | 0.27±1.20 | 0.63 |
|  | 11 | 0.51±1.62 | -0.07±1.68 | 0.27 |  | 11 | -0.51±4.82 | 0.37±1.20 | 0.43 |
|  | 12 | 0.35±1.11 | -0.12±1.77 | 0.30 |  | 12 | -0.01±2.13 | 0.61±1.77 | 0.30 |
|  | 13 | 0.46±1.08 | -0.43±1.31 | **0.02*** |  | 13 | 0.56±2.06 | 0.44±1.46 | 0.83 |
|  | 14 | 0.33±1.12 | 0.47±1.06 | 0.67 |  | 14 | 0.52±1.42 | 0.41±1.33 | 0.78 |
|  | 15 | 0.59±1.91 | 0.25±1.46 | 0.52 |  | 15 | 0.58±1.87 | 0.06±1.84 | 0.35 |
| 3^rd^  rest | 1 | -0.08±0.54 | 0.01±1.25 | 0.78 | Task3  (Color-Word) | 1 | 0.18±1.24 | 0.07±1.50 | 0.80 |
|  | 2 | 0.17±1.61 | -0.32±1.31 | 0.72 |  | 2 | 0.58±1.09 | 0.29±1.99 | 0.55 |
|  | 3 | 0.15±0.92 | 0.11±0.98 | 0.90 |  | 3 | 0.43±0.89 | 0.42±1.13 | 0.97 |
|  | 4 | 0.18±0.99 | -0.27±1.11 | 0.16 |  | 4 | 0.57±0.94 | -0.02±0.88 | **0.04*** |
|  | 5 | 0.36±1.44 | -0.32±0.94 | 0.07 |  | 5 | 0.44±0.92 | 0.04±1.00 | 0.17 |
|  | 6 | 0.55±1.32 | -0.20±0.63 | **0.21*** |  | 6 | 0.48±1.35 | 0.28±0.94 | 0.56 |
|  | 7 | 0.38±1.18 | -0.13±0.87 | 0.11 |  | 7 | 0.51±1.13 | -0.15±0.85 | **0.03*** |
|  | 8 | 0.11±0.63 | -0.33±0.79 | **0.05*** |  | 8 | 0.43±1.57 | 0.02±0.55 | 0.24 |
|  | 9 | -0.05±1.47 | -0.14±1.23 | 0.84 |  | 9 | 0.19±3.03 | 0.18±1.20 | 0.99 |
|  | 10 | -0.20±1.53 | -0.53±1.11 | 0.45 |  | 10 | 0.57±1.98 | -0.06±0.58 | 0.17 |
|  | 11 | 0.76±1.89 | -0.34±1.49 | **0.05*** |  | 11 | -0.08±1.43 | 0.07±1.21 | 0.72 |
|  | 12 | 0.17±0.81 | 0.01±0.99 | 0.55 |  | 12 | -0.55±2.23 | 0.37±0.95 | 0.09 |
|  | 13 | 0.01±1.12 | -0.72±1.90 | 0.12 |  | 13 | 0.42±1.18 | 0.13±0.80 | 0.34 |
|  | 14 | 0.07±1.47 | 0.12±0.74 | 0.88 |  | 14 | 0.27±1.16 | -0.10±1.08 | 0.27 |
|  | 15 | 0.13±0.96 | 0.08±1.37 | 0.89 |  | 15 | 0.60±1.96 | 0.29±1.07 | 0.52 |

Data are the mean±SD (10^-3^). ADHD, attention deficit hyperactivity disorder; SD, standard deviation. *Statistically significant at *p* < 0.05, based on Bonferroni correction for multiple comparisons calculated using the NIRSIT Lite Analysis Tool.

**Supplementary Table 2.** Hemispheric asymmetry score of the prefrontal cortex in HbO and HbR between groups

| **Section** | | **Medicated ADHD**  **(n=20)** | **Unmedicated ADHD**  **(n=18)** | ***p*** | ***p***^†^ |
| --- | --- | --- | --- | --- | --- |
| **HbO** | | | | | |
| Rest | 1 | 1.53±7.67 | -0.22±1.59 | 0.35 | 0.39 |
|  | 2 | 1.19±4.56 | 0.14±1.01 | 0.35 | 0.39 |
|  | 3 | 0.68±1.98 | -0.33±1.25 | 0.72 | 0.16 |
| Task | 1 | 0.78±3.00 | 1.86±13.34 | 0.73 | 0.35 |
|  | 2 | 0.32±3.30 | -0.11±4.79 | 0.75 | 0.53 |
|  | 3 | 0.03±1.43 | -1.00±3.83 | 0.27 | 0.54 |
| **HbR** | | | | | |
| Rest | 1 | -0.24±1.25 | -0.18±1.39 | 0.88 | 0.96 |
|  | 2 | 0.66±2.75 | 0.60±4.85 | 0.97 | 0.85 |
|  | 3 | 1.19±4.04 | 0.36±1.20 | 0.40 | 0.41 |
| Task | 1 | 0.72±3.99 | 0.01±4.80 | 0.62 | 0.43 |
|  | 2 | 0.67±1.97 | -0.37±1.16 | 0.06 | 0.13 |
|  | 3 | 0.05±1.43 | -0.67±2.02 | 0.21 | 0.26 |

Data are the mean±SD. The asymmetry score was calculated as the average of channels 9-15 for the right hemisphere and channels 1-7 for the left hemisphere to subjects with no channels rejected and results in all channels. HbO, oxygenated hemoglobin; ADHD, attention deficit/hyperactivity disorder; ARS, ADHD rating scale; ATA, advanced test of attention, RT, response time; SD, standard deviation. ^†^Hemispheric asymmetry score was adjusted for sex, age, and handedness.

**Supplementary Table 3.** Interaction effect between clinical scores and HbO of channel 9 in the 1^st^ rest

| **psychiatric assessment** | **subscale/subset** | **Medicated ADHD**  **(n=23)** | | **Unmedicated ADHD**  **(n=22)** | | ***p*** | ***p***^†^ |
| --- | --- | --- | --- | --- | --- | --- | --- |
|  |  | *r* | *p* | *r* | *p* |  |  |
| ARS | inattention | 0.302 | 0.09 | 0.100 | 0.66 | 0.44 | 0.53 |
|  | Hyperactivity-impulsivity | -0.151 | 0.49 | 0.016 | 0.94 | 0.54 | 0.57 |
|  | Total | 0.100 | 0.65 | 0.060 | 0.79 | 0.83 | 0.87 |
| ATA Visual | Omission error | -0.175 | 0.43 | 0.011 | 0.96 | 0.52 | 0.64 |
|  | Commission error | -0.324 | 0.13 | 0.186 | 0.41 | 0.09 | 0.07 |
|  | RT | -0.249 | 0.03 | 0.172 | 0.04***** | 0.18 | 0.20 |
|  | RTV | -0.314 | 0.15 | 0.390 | 0.07 | **0.02*** | **0.02*** |
| ATA Auditory | Omission error | 0.150 | 0.50 | 0.244 | 0.28 | 0.83 | 0.93 |
|  | Commission error | 0.051 | 0.82 | 0.303 | 0.17 | 0.49 | 0.35 |
|  | RT | 0.195 | 0.37 | 0.134 | 0.55 | 0.64 | 0.57 |
|  | RTV | 0.096 | 0.66 | 0.261 | 0.24 | 0.65 | 0.79 |
| SCWT | Word | 0.212 | 0.33 | -0.025 | 0.91 | 0.37 | 0.33 |
|  | Color | 0.102 | 0.65 | 0.191 | 0.40 | 0.83 | 0.92 |
|  | Color-Word | 0.205 | 0.35 | -0.176 | 0.44 | 0.23 | 0.30 |
|  | Interference | 0.113 | 0.61 | -0.424 | 0.01***** | 0.12 | 0.20 |

Data are the mean±SD. HbO, oxygenated hemoglobin; ADHD, attention deficit/hyperactivity disorder; ARS, ADHD rating scale; ATA, advanced test of attention; SCWT, stroop color-word test; RT, response time; RTV, response time variability. *Statistically significant at *p* < 0.05. ^†^Adjusted for sex, age, handedness, and medication type (atomoxetine, methylphenidate, or both).

**Supplementary Table 4.** Simple effect analysis of ATA visual RTV and group interaction on HbO in channel 9

| **Group** | **β** | **SE** | **95% CI** | **df** | ***p*** |
| --- | --- | --- | --- | --- | --- |
| Medicated ADHD | 0.025 | 0.145 | -0.005 to 0.054 | 41 | 0.10 |
| Unmedicated ADHD | -0.021 | 0.127 | -0.047 to 0.005 | 41 | 0.11 |

β, SE, and CI values are presented in units of ×10⁻³. ATA, advanced test of attention; RTV, response time variability; HbO, oxygenated hemoglobin; SE, standard error; CI, confidence interval; df, degrees of freedom.

**Supplementary Table 5.** Interaction effect between clinical scores and HbO of channel 13 and 14 in task 2 (Color test)

| **channel** | **test** | **subset** | **Medicated ADHD**  **(n=23)** | | **Unmedicated ADHD**  **(n=22)** | | ***p*** | ***p***^†^ |
| --- | --- | --- | --- | --- | --- | --- | --- | --- |
|  |  |  | *r* | *p* | *r* | *p* |  |  |
| 13 | ARS | Inattention | 0.113 | 0.61 | -0.413 | 0.06 | 0.07 | 0.13 |
|  |  | Hyperactivity-impulsivity | -0.310 | 0.15 | -0.481 | **0.02*** | 0.63 | 0.70 |
|  |  | Total | -0.103 | 0.64 | -0.480 | **0.02*** | 0.23 | 0.32 |
|  | ATA Visual | Omission error | 0.230 | 0.29 | -0.004 | 0.99 | 0.47 | 0.87 |
|  |  | Commission error | 0.413 | 0.05 | -0.156 | 0.49 | 0.07 | 0.08 |
|  |  | RT | -0.337 | 0.12 | 0.074 | 0.74 | 0.22 | 0.26 |
|  |  | RTV | -0.063 | 0.78 | -0.234 | 0.30 | 0.51 | 0.48 |
|  | ATA Auditory | Omission error | -0.115 | 0.60 | 0.257 | 0.25 | 0.22 | 0.46 |
|  |  | Commission error | 0.067 | 0.76 | -0.002 | 0.99 | 0.83 | 0.65 |
|  |  | RT | 0.099 | 0.65 | 0.208 | 0.35 | 0.82 | 0.79 |
|  |  | RTV | 0.042 | 0.85 | 0.007 | 0.98 | 0.92 | 0.42 |
|  | SCWT | Word | 0.412 | **0.01*** | 0.422 | 0.05 | 0.82 | 0.77 |
|  |  | Color | 0.209 | 0.34 | 0.006 | 0.98 | 0.55 | 0.95 |
|  |  | Color-Word | 0.253 | 0.24 | 0.214 | 0.34 | 0.97 | 0.60 |
|  |  | Interference | 0.027 | 0.90 | 0.236 | 0.29 | 0.52 | 0.50 |
| 14 | ARS | inattention | 0.046 | 0.84 | -0.414 | 0.06 | 0.13 | 0.18 |
|  |  | Hyperactivity-impulsivity | -0.199 | 0.36 | -0.170 | 0.45 | 0.84 | 0.75 |
|  |  | total | -0.08 | 0.71 | -0.306 | 0.17 | 0.54 | 0.66 |
|  | ATA Visual | Omission error | 0.067 | 0.76 | -0.109 | 0.63 | 0.58 | 0.35 |
|  |  | Commission error | 0.075 | 0.74 | -0.123 | 0.59 | 0.53 | 0.59 |
|  |  | RT | -0.406 | 0.06 | -0.121 | 0.59 | 0.40 | 0.58 |
|  |  | RTV | -0.167 | 0.45 | -0.319 | 0.15 | 0.56 | 0.44 |
|  | ATA Auditory | Omission error | -0.027 | 0.90 | 0.002 | 0.99 | 0.93 | 0.92 |
|  |  | Commission error | 0.112 | 0.61 | -0.136 | 0.55 | 0.43 | 0.58 |
|  |  | RT | 0.065 | 0.77 | 0.163 | 0.47 | 0.86 | 0.85 |
|  |  | RTV | 0.145 | 0.51 | -0.183 | 0.41 | 0.29 | 0.14 |
|  | SCWT | Word | 0.355 | 0.10 | -0.016 | 0.94 | 0.17 | 0.14 |
|  |  | Color | -0.048 | 0.83 | -0.149 | 0.51 | 0.74 | 0.52 |
|  |  | Color-Word | 0.149 | 0.50 | -0.253 | 0.26 | 0.19 | 0.20 |
|  |  | Interference | 0.256 | 0.24 | -0.04 | 0.85 | 0.31 | 0.54 |

Data are the mean±SD. HbO, oxygenated hemoglobin; ADHD, attention deficit/hyperactivity disorder; ARS, ADHD rating scale; ATA, advanced test of attention; RT, response time; RTV, response time variability; SCWT, stroop color-word test. *Statistically significant at *p* < 0.05. ^†^Adjusted for sex, age, handedness, and medication type (atomoxetine, methylphenidate, or both).

**Supplementary Table 6.** Differences in mean global and local network properties between medicated and unmedicated ADHD children

| **network** | **section**  **(channel)** | **network**  **properties** | **Medicated ADHD**  **(n=23)** | **Unmedicated ADHD**  **(n=22)** | ***p^†^*** | ***p^‡^*** |
| --- | --- | --- | --- | --- | --- | --- |
| Global network | 1^st^  rest | Ge | 0.44±0.06 | 0.43±0.05 | 0.65 | 0.86 |
|  |  | C_p_ | 0.64±0.09 | 0.65±0.09 | 0.86 | 0.86 |
|  | Task 2 Color | Ge | 0.43±0.06 | 0.46±0.06 | 0.38 | 0.76 |
|  |  | C_p_ | 0.66±0.07 | 0.61±0.07 | **0.03*** | 0.13 |
| Local  network | 1^st^  rest (9) | NC_p_ | 0.84±0.22 | 0.78±0.20 | 0.16 | 0.47 |
|  |  | DC_w_ | 3.80±2.19 | 5.05±1.61 | **0.03*** | 0.19 |
|  | Task 2 Color  (13) | NC_p_ | 0.83±0.19 | 0.82±0.13 | 0.98 | 0.98 |
|  |  | DC_w_ | 4.30±2.32 | 4.68±2.46 | 0.48 | 0.95 |
|  | Task 2 Color  (14) | NC_p_ | 0.81±0.17 | 0.81±0.14 | 0.67 | 0.98 |
|  |  | DC_w_ | 5.20±1.79 | 5.05±2.07 | 0.88 | 0.98 |

Data are the mean±SD. Ge, global efficiency; C_p_, clustering coefficient; NC_p_, nodal clustering coefficient; DC_w_, weighted degree centrality. *Statistically significant at *p* < 0.05. **^†^**Adjusted for age, sex. **^‡^**FDR corrected *p*.

**Supplementary Table 7**. Comparison of brain volumes between the groups

| **Volume measures** | **Medicated**  **ADHD**  **(n=17)** | **Unmedicated**  **ADHD**  **(n=14)** | ***p*** |
| --- | --- | --- | --- |
| **Absolute volumes (mm^3^)** |  |  |  |
| **Gross volume** |  |  |  |
| Ventricle | 15919.06±5937.01 | 14295.21±6362.66 | 0.47 |
| Cerebral white matter | 414273.29±52848.66 | 429373.07±58912.13 | 0.46 |
| Subcortical gray matter | 61480.41±5257.32 | 61359.07±4640.87 | 0.95 |
| total gray matter | 713408.81±83238.30 | 737986.80±61047.78 | 0.37 |
| Cortex | 550611.57±58987.87 | 568351.30±51131.55 | 0.38 |
| **Left cortex** | 276042.11±28822.43 | 284329.27±26437.12 | 0.42 |
| **Right cortex** | 274569.46±30255.12 | 284022.02±24923.22 | 0.36 |
| **Left cerebellum volumes** |  |  |  |
| Cerebellum white matter | 13587.74±1546.21 | 13931.74±2094.93 | 0.60 |
| Cerebellum cortex | 55669.85±5311.91 | 54483.37±6706.70 | 0.59 |
| **Right cerebellum volumes** |  |  |  |
| Cerebellum white matter | 12961.60±1498.97 | 13221.84±1855.43 | 0.67 |
| Cerebellum cortex | 55871.46±5131.10 | 54127.86±7202.91 | 0.44 |
| **Left subcortex volumes** |  |  |  |
| Thalamus | 7776.74±827.13 | 7901.74±566.28 | 0.64 |
| Caudate | 3827.16±423.26 | 3664.39±497.70 | 0.33 |
| Putamen | 5749.53±526.92 | 5882.61±528.11 | 0.49 |
| Pallidum | 1923.11±289.25 | 1933.60±267.12 | 0.92 |
| Hippocampus | 4135.47±389.71 | 3995.43±430.58 | 0.35 |
| Amygdala | 1796.05±271.48 | 1748.68±208.46 | 0.60 |
| **Right subcortex volumes** |  |  |  |
| Thalamus | 7584.43±777.20 | 7654.72±598.97 | 0.78 |
| Caudate | 4084.06±490.99 | 3885.28±501.11 | 0.28 |
| Putamen | 5809.68±531.31 | 5951.14±456.51 | 0.44 |
| Pallidum | 1841.62±271.52 | 1880.21±287.35 | 0.70 |
| Hippocampus | 4167.49±377.15 | 4187.11±365.67 | 0.89 |
| Amygdala | 1892.50±248.78 | 1855.06±215.86 | 0.66 |
| **Relative to ICV (%)** |  |  |  |
| **Gross volume** |  |  |  |
| Ventricle | 1.07±0.39 | 0.96±0.39 | 0.44 |
| Cerebral white matter | 27.77±2.28 | 28.88±2.33 | 0.19 |
| Subcortical gray matter | 11.25±1.23 | 10.86±1.11 | 0.37 |
| Total gray matter | 47.92±4.58 | 49.95±4.57 | 0.23 |
| Cortex | 47.39±1.91 | 47.75±2.69 | 0.67 |
| **Left cortex** | 18.57±1.77 | 19.26±2.19 | 0.34 |
| **Right cortex** | 18.47±1.86 | 19.25±2.14 | 0.30 |
| **Left cerebellum volumes** |  |  |  |
| Cerebellum white matter | 0.91±0.09 | 0.94±0.09 | 0.46 |
| Cerebellum cortex | 3.75±0.33 | 3.67±0.28 | 0.49 |
| **Right cerebellum volumes** |  |  |  |
| Cerebellum white matter | 0.87±0.09 | 0.89±0.07 | 0.57 |
| Cerebellum cortex | 3.76±0.35 | 3.64±0.28 | 0.30 |
| **Left subcortex volumes** |  |  |  |
| Thalamus | 0.52±0.03 | 0.35±0.05 | 0.38 |
| Caudate | 0.26±0.02 | 0.25±0.04 | 0.39 |
| Putamen | 0.39±0.03 | 0.40±0.04 | 0.32 |
| Pallidum | 0.13±0.01 | 0.13±0.02 | 0.80 |
| Hippocampus | 0.28±0.02 | 0.27±0.02 | 0.24 |
| Amygdala | 0.12±0.01 | 0.12±0.01 | 0.60 |
| **Right subcortex volumes** |  |  |  |
| Thalamus | 0.51±0.02 | 0.52±0.04 | 0.42 |
| Caudate | 0.27±0.03 | 0.26±0.03 | 0.29 |
| Putamen | 0.39±0.03 | 0.40±0.04 | 0.27 |
| Pallidum | 0.12±0.01 | 0.13±0.01 | 0.56 |
| Hippocampus | 0.28±0.03 | 0.28±0.02 | 0.78 |
| Amygdala | 0.13±0.01 | 0.13±0.01 | 0.71 |

Data are the mean±SD. ADHD, attention deficit hyperactivity disorder; ICV, intracranial volume.

**Supplementary Table 8**. Comparison of brain volumes among the groups based on medication type

| **Volume measures** | **Methylphenidate only**  **(n=8)** | **Atomoxetine only**  **(n=4)** | **Both**  **(N=5)** | ***p*** |
| --- | --- | --- | --- | --- |
| **Absolute volumes (mm^3^)** |  |  |  |  |
| **Gross volume** |  |  |  |  |
| Ventricle | 12751.63±4139.28 | 16728.25±7269.12 | 20339.60±5121.68 | 0.07 |
| Cerebral white matter | 408107.25±58963.41 | 407996.25±53468.18 | 429160.60±50508.53 | 0.78 |
| Subcortical gray matter | 61964.00±4710.97 | 60518.50±7683.43 | 61476.20±5103.93 | 0.92 |
| total gray matter | 705086.88±80313.06 | 727574.64±60338.14 | 715391.22±115550.96 | 0.92 |
| Cortex | 531858.26±71806.32 | 555105.64±38112.62 | 577021.62±47012.26 | 0.43 |
| **Left cortex** | 267629.80±35006.59 | 277585.21±19385.65 | 288267.33±23786.24 | 0.48 |
| **Right cortex** | 264228.46±36876.50 | 277520.43±18781.89 | 288754.30±23303.30 | 0.38 |
| **Left cerebellum volumes** |  |  |  |  |
| Cerebellum white matter | 13725.39±1454.04 | 12675.98±2431.64 | 14096.90±484.99 | 0.39 |
| Cerebellum cortex | 55448.61±4563.94 | 56390.93±9579.36 | 55446.96±2628.79 | 0.96 |
| **Right cerebellum volumes** |  |  |  |  |
| Cerebellum white matter | 13166.83±1422.82 | 12292.18±2478.94 | 13168.78±499.96 | 0.62 |
| Cerebellum cortex | 56192.86±3818.52 | 56231.03±9526.75 | 55069.56±3253.75 | 0.93 |
| **Left subcortex volumes** |  |  |  |  |
| Thalamus | 7813.69±886.77 | 7740.20±898.66 | 7746.84±787.84 | 0.99 |
| Caudate | 3927.64±346.00 | 3649.75±685.22 | 3808.32±320.72 | 0.59 |
| Putamen | 5752.00±553.11 | 5657.73±672.32 | 5819.02±470.85 | 0.91 |
| Pallidum | 2034.25±293.88 | 1823.73±299.08 | 1824.78±264.15 | 0.35 |
| Hippocampus | 4064.65±197.70 | 4308.03±487.84 | 4110.74±565.71 | 0.62 |
| Amygdala | 1768.04±205.16 | 1725.43±236.93 | 1897.38±399.55 | 0.62 |
| **Right subcortex volumes** |  |  |  |  |
| Thalamus | 7654.45±701.21 | 7488.98±1163.07 | 7548.76±720.67 | 0.94 |
| Caudate | 4163.86±483.30 | 3992.63±709.90 | 4029.52±390.82 | 0.83 |
| Putamen | 5798.08±565.02 | 5716.93±688.24 | 5902.44±442.27 | 0.88 |
| Pallidum | 1898.80±271.82 | 1830.25±374.51 | 1759.22±212.72 | 0.69 |
| Hippocampus | 4050.84±211.46 | 4153.43±417.80 | 4365.40±532.37 | 0.37 |
| Amygdala | 1884.00±275.77 | 1863.98±216.10 | 1928.92±277.18 | 0.93 |
| **Relative to ICV (%)** |  |  |  |  |
| **Gross volume** |  |  |  |  |
| Ventricle | 0.85±0.28 | 1.11±0.44 | 1.37±0.35 | 0.06 |
| Cerebral white matter | 27.46±3.06 | 27.37±0.60 | 28.58±1.17 | 0.66 |
| Subcortical gray matter | 11.81±1.58 | 10.87±0.65 | 10.66±0.41 | 0.21 |
| Total gray matter | 47.59±5.17 | 49.07±3.08 | 47.52±5.30 | 0.86 |
| Cortex | 46.68±2.20 | 48.05±1.95 | 47.99±1.17 | 0.38 |
| **Left cortex** | 18.06±2.30 | 18.75±1.50 | 19.23±0.73 | 0.53 |
| **Right cortex** | 18.02±2.63 | 18.75±1.43 | 19.27±0.73 | 0.54 |
| **Left cerebellum volumes** |  |  |  |  |
| Cerebellum white matter | 0.93±0.09 | 0.85±0.08 | 0.94±0.07 | 0.22 |
| Cerebellum cortex | 3.74±0.31 | 3.78±0.33 | 3.73±0.44 | 0.98 |
| **Right cerebellum volumes** |  |  |  |  |
| Cerebellum white matter | 0.89±0.10 | 0.82±0.10 | 0.88±0.08 | 0.50 |
| Cerebellum cortex | 3.80±0.33 | 3.77±0.36 | 3.70±0.43 | 0.89 |
| **Left subcortex volumes** |  |  |  |  |
| Thalamus | 0.53±0.04 | 0.52±0.03 | 0.52±0.03 | 0.88 |
| Caudate | 0.27±0.02 | 0.24±0.03 | 0.25±0.02 | 0.40 |
| Putamen | 0.39±0.02 | 0.38±0.04 | 0.39±0.03 | 0.91 |
| Pallidum | 0.14±0.01 | 0.12±0.02 | 0.12±0.01 | 0.11 |
| Hippocampus | 0.28±0.02 | 0.29±0.01 | 0.27±0.03 | 0.60 |
| Amygdala | 0.12±0.01 | 0.12±0.01 | 0.13±0.02 | 0.40 |
| **Right subcortex volumes** |  |  |  |  |
| Thalamus | 0.52±0.02 | 0.50±0.03 | 0.50±0.02 | 0.50 |
| Caudate | 0.28±0.03 | 0.27±0.03 | 0.27±0.02 | 0.70 |
| Putamen | 0.39±0.02 | 0.39±0.04 | 0.39±0.03 | 0.88 |
| Pallidum | 0.13±0.01 | 0.12±0.02 | 0.12±0.01 | 0.43 |
| Hippocampus | 0.27±0.02 | 0.28±0.01 | 0.29±0.04 | 0.47 |
| Amygdala | 0.13±0.01 | 0.13±0.01 | 0.13±0.02 | 0.93 |

Data are the mean±SD. ICV, intracranial volume.

**Supplementary Table 9**. Comparison of asymmetry scores between the groups

| **volume measures** | **ADHD with**  **medication**  **(n=17)** | **ADHD without medication**  **(n=14)** | ***p***^†^ |
| --- | --- | --- | --- |
| **Gross volume** |  |  |  |
| Cortex | 0.22±0.40 | 0.12±0.32 | 0.45 |
| Cerebellum white matter | 2.71±0.87 | 2.12±0.76 | 0.05 |
| Cerebellum cortex | 0.26±0.65 | -0.17±0.81 | 0.11 |
| Thalamus | 1.48±0.60 | 1.32±0.67 | 0.48 |
| Caudate | -2.84±1.28 | -3.41±1.01 | 0.19 |
| Putamen (striatum) | -0.47±0.57 | -0.70±0.45 | 0.24 |
| Pallidum | 1.92±0.63 | 1.75±0.58 | 0.46 |
| Hippocampus | -1.07±0.70 | -1.62±0.65 | **0.03*** |
| Amygdala | -3.40±2.64 | -2.08±0.96 | 0.09 |
| **Subthalamus volumes** |  |  |  |
| **Medial** |  |  |  |
| Mediodorsal lateral parvocellular | -2.20±2.31 | -0.67±2.30 | 0.08 |
| Mediodorsal medial magnocellular | -1.24±2.03 | -0.41±1.77 | 0.24 |
| Reuniens (medial ventral) | -4.47±3.23 | -4.46±1.99 | 1.00 |
| Paratenial | -2.30±1.09 | -3.24±1.20 | **0.03*** |
| **Intralaminar** |  |  |  |
| Central medial | 0.67±2.08 | -0.67±0.87 | 0.23 |
| Centromedian | 0.80±0.99 | 0.01±1.02 | **0.04*** |
| Central lateral | 0.63±2.08 | -0.89±2.95 | 0.19 |
| Paracentral | -4.28±2.61 | -6.33±2.74 | **0.04*** |
| Parafascicular | -1.51±3.03 | -2.43±1.28 | 0.30 |

Data are the mean±SD. *Statistically significant at *p* < 0.05. ^†^Absolute volumes were adjusted for sex, age, handedness, and total volume excluded ventricle (gross volume) or whole thalamus volume (subcortex volume).
